# Lack of association between serotonin transporter 5-HTT gene polymorphism and endometriosis in an Italian patient population

**DOI:** 10.1186/1477-5751-13-12

**Published:** 2014-06-12

**Authors:** Francesca Megiorni, Serena Resta, Deliar Yazdanian, Gabriele Cavaggioni, Claudia Lia, Pierluigi Benedetti Panici, Antonio Pizzuti, Maria Grazia Porpora

**Affiliations:** 1 Department of Experimental Medicine, Policlinico Umberto I, Sapienza University of Rome, Viale Regina Elena 324, 00161 Rome, Italy; 2 Department of Gynaecology, Obstetrics and Urology, Policlinico Umberto I, Sapienza University of Rome, Viale del Policlinico 155, 00161 Rome, Italy; 3 Neurology and Psychiatry, U.O.D. Psychotherapy, Policlinico Umberto I, Via Casal dei Pazzi 16, 00156 Rome, Italy

**Keywords:** Endometriosis, Serotonin transporter gene (5-HTT) L/S polymorphism, Case–control study

## Abstract

**Background:**

The aim of this study was to determine whether the serotonin transporter gene (5-HTT), a key component in the control of the serotonergic system, is associated with endometriosis in an Italian population.

**Findings:**

A case–control study, comprising 137 Italian patients with surgically confirmed endometriosis and 120 healthy controls, was carried out. 5-HTT genotypes (LL, SL and SS) were obtained by polymerase chain reaction and gel electrophoresis analysis. We found no overall difference in genotypic and allelic distributions of the 5-HTT gene between cases and controls.

**Conclusions:**

Our results suggest that the 5-HTT L/S promoter polymorphism is not associated with susceptibility to endometriosis in the studied Italian patients.

## Findings

### Introduction

Endometriosis is a gynecological condition characterized by the presence of ectopic endometrial tissue (endometrial glands and stroma) outside the uterus associated with pelvic pain and infertility [1]. The disease affects 6-10% of women in reproductive age with or without pelvic pain and more than 30% of infertile women [2-4]. Endometriosis has a multifactorial inheritance: environmental pollutants, particularly dioxins and polychlorinated biphenyls (PCBs) and genetic predisposition have been suggested to concur to the onset and progression of this disease [5-8]. Endometriosis varies from a mild disease with only peritoneal lesions to a severe form involving both ovaries associated with infiltrating foci and extensive adhesions. Women with endometriosis may have a range of pelvic and abdominal pain symptoms, including dysmenorrhoea, dyspareunia, non-menstrual (chronic) pelvic pain, pain at ovulation, dyschezia and dysuria [9,10]. Endometriosis and its symptoms tend to recur after treatment [11]. Pain symptoms significantly vary among patients and do not always correlate with the severity of endometriosis [9,12,13] suggesting that other factors, such as psychological factors, altered stress response and emotional factors may influence the perception of pain [14]. Alterations in the immune system, such as an increase in activated peritoneal immune cells and pro-inflammatory factors, seem to be involved in the pathogenesis of endometriosis [15]. Serotonin (5-hydroxytryptamine; 5-HT) is a neurotransmitter that participates in many physiological processes such as sleep, appetite, pain perception, hormone secretion, and sexual behavior [16]. 5-HT is also able to regulate important functions outside of the CNS (central nervous system), such as vasoconstriction, proliferation and insulin secretion, and can trigger inflammatory mechanisms [17,18]. Serotonin transporter (5-HTT) uptakes 5-HT from the synaptic cleft and plays a critical role in the termination of the serotonergic system, such as stress response. Recent findings have suggested that a 5-HTT-dependent mechanism is also involved in the pathogenesis of autoimmune and chronic inflammatory diseases [19]. Human 5-HTT gene maps on chromosome 17q11.1-12 and its expression is regulated at the transcriptional level by the presence/absence of a stretch of 44 nucleotides located in the promoter region, leading to two different variants referred to as L (Long) and S (Short). S allele is associated with low levels of 5-HTT mRNA and therefore with a reduced serotonin re-uptake. SL or SS subjects have a greater tendency to stress compared to LL homozygous individuals [20]. Therefore, by regulating the magnitude and duration of serotonergic and neuroendocrine responses, 5-HTT expression is central to the fine-tuning of both brain serotonergic neurotransmission and immunomodulatory effects.

The aim of the present study was to assess the role of genetic factors in the etiopathogenesis of endometriosis linked to the genotype of the serotonin transporter gene regulatory region compared with healthy women.

### Materials and methods

#### *Patients and controls*

In total 137 unrelated Caucasian patients from peninsular Italy, ranging from unskilled workers to university graduates, were included in the study. All had endometriosis and mean age was 37.1 ± 7.7 years. Diagnosis of endometriosis was achieved by laparoscopy and histologic analysis. As healthy controls, 120 women from the same ethnic area were enrolled with mean age 35.2 ± 11.2 years and pelvic examination and transvaginal ultrasound were performed and all results were normal. Laparoscopy was not performed systematically, but when available, it showed normal results. All patients were recruited from the Department of Gynecology and Obstetrics – Policlinico Umberto I, “Sapienza” University of Rome, Italy. The Institutional Review Board of the University Hospital Azienda Policlinico Umberto I approved the study protocol and all subjects provided their informed consent.

#### *DNA extraction and 5-HTTLPR genotyping*

**Figure 1:**
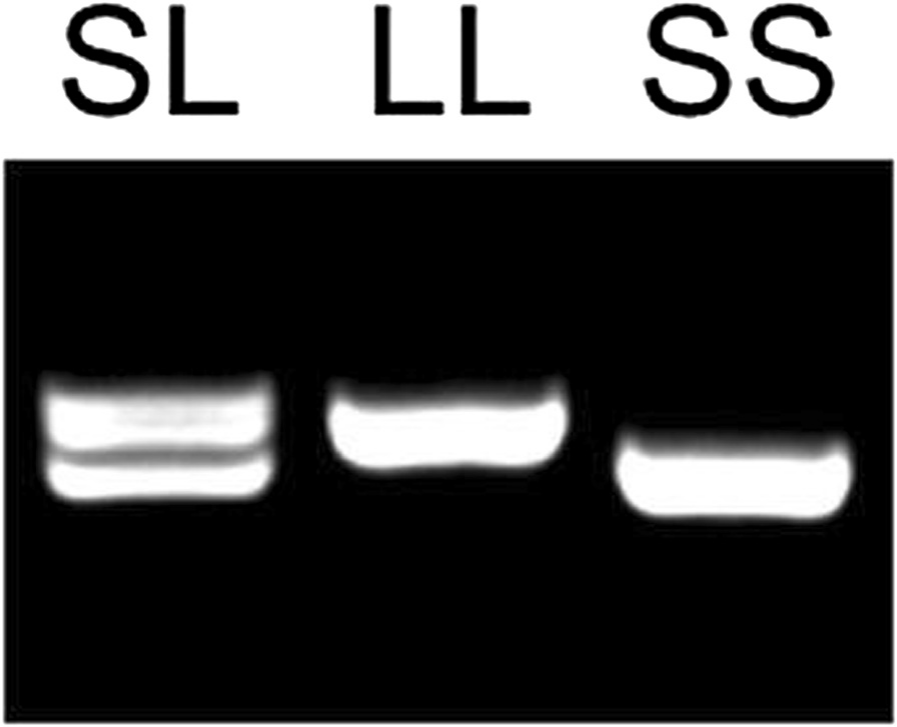
**Genotyping of 5-HTT L/S polymorphism using PCR/agarose gel electrophoresis.** Example of SL, LL and SS genotypes.

Genomic DNA was extracted from 5 ml peripheral venous blood using a salting-out procedure [21]. The L/S (long allele-L/short allele-S) polymorphism in the promoter region of the 5-HTT gene consisting of an insertion/deletion (long allele-L/short allele-S) of 44 base pair (bp) was amplified by polymerase chain reaction (PCR) using 5-HTT-forward: 5’-GGCGTTGCCGCTCTGAATGC-3’ and 5-HTT-reverse: 5’-GAGGGACTGAGCTGGACAACCAC-3’ primers. PCR conditions were as follows: denaturation for 4 min at 95°C, 35 cycles of 45 sec at 95°C, 45 sec at 67°C and 90 sec at 72°C, followed by 5 min at 72°C. PCR products were separated in 2% agarose gels with ethidium bromide and visualized under Ultraviolet (UV) light. Band patterns of the different combinations of S (485 bp) and L alleles (529 bp) are shown in Figure 1.

#### *Statistical analysis*

All basic statistical analyses were performed with the Statistical Product and Service Solutions software (SPSS) version 17.0 WIN program (SPSS, Chicago, Illinois). The *χ*2 or Fisher’s exact tests were used to compare differences between categorical variables. A p-value <0.05 was considered statistically significant. 5-HTT genotype distribution was evaluated for Hardy-Weinberg equilibrium by GenAlEx 6 (Genetic analysis in Excel) software [22].

### Results

To investigate whether the 5-HTT L/S polymorphism was associated with endometriosis in Italian female population, we compared 137 unrelated patients with 120 healthy women. Genotype distribution resulted in Hardy-Weinberg equilibrium in both cases and controls (data not shown). Table 1 summarizes the allele and genotype frequencies of 5-HTT in affected and healthy women. A strong predominance of L allele was evident in both groups (57.3% in cases and 56.7% in controls), whereas S allele was present less frequently (42.7% in cases and 43.3% in controls). The distribution of the three different genotypes did not show any significant differences between patients and control subjects (Table 1). In an inheritance model in which S is the dominant allele and the risk factor for endometriosis, the prevalence of SL heterozygotes and SS homozygotes, collectively considered, resulted comparable in affected women and controls (65.0% vs. 65.8%). In all, our results indicate that the presence of the 5-HTT S variant is not associated with the risk of endometriosis.

**Table 1:** **Allele and genotype frequencies of the *5-HTT *** **gene polymorphism in patients with endometriosis and controls**

***5-HTT***	**Patients (137)**		**Controls (120)**	
	**n**	**%**	**n**	**%**
**Allele**				
**S**	117	(42.7%)	104	(43.3%)
**L**	157	(57.3%)	136	(56.7%)
**Genotype**				
**SS**	28	(20.5%)	25	(20.8%)
**SL**	61	(44.5%)	54	(45.0%)
**LL**	48	(35.0%)	41	(34.2%)
**Dominant model**				
**SS + SL**	89	(65.0%)	79	(65.8%)
**LL**	48	(35.0%)	41	(34.2%)

The genetic model was considered dominant in the hypothesis that the S variant is a risk factor for the disease.

#### *Comment*

The aim of this study was to elucidate a possible role of the functional L/S polymorphism in the serotonin transporter gene (5-HTT) in patients with endometriosis since the axis 5-HT/5-HTT has been correlated with alterations of both immune and stress response [19,20]. The results of our case–control study failed to show an association between the 5-HTT L/S variants and endometriosis susceptibility. Furthermore, this gene polymorphism of the serotoninergic system seems to be not associated with clinical features of endometriosis, but this was possible due to the small number of patients available in the present study. Our analysis cannot rule out the possible involvement of other genes that have been related to reactions to stress and/or inflammation, such as 5-HT2A receptor 102 T/C polymorphisms, suggesting the serotonergic system may play a role in the pathogenesis of endometriosis. Additional studies including larger number of patients and controls are required to elucidate candidate genes that are likely to be involved in both the sensory and affective components of pain linked to endometriosis.

In conclusion, this study shows that the 5-HTT L/S promoter polymorphism seems not to confer susceptibility to endometriosis in the Italian population.

## Abbreviations

5-HTT: Serotonin transporter; 5-HT: Serotonin; PCBs: Polychlorobiphenyls; CNS: Central nervous system; L: Long; S: Short; L/S: Long allele-L/short allele-S; Bp: Base pair; UV: Ultraviolet; PCR: Polymerase chain reaction; SPSS: Statistical product and service solutions software; GenAlEx: Genetic analysis in excel.

## Competing interests

The authors declare that they have no competing interests.

## Authors’ contributions

MGP, FM and PBP conceived the study and participated in its design. FM and AP carried out the molecular genetic studies and participated in the sequence alignment. FM, CL, SR and DY performed the statistical analysis. MGP, FM, SR, DY, GC and CL participated in the analysis and interpretation of data. DY, GC, CL and AP collected the data. MGP, FM, SR, DY, AP, and PBP wrote the paper. All authors read and approved the final manuscript.
